# Effect of Disease Extent on Leucine‐Rich α2‐Glycoprotein as a Marker for Endoscopic Mucosal Healing in Patients With Ulcerative Colitis

**DOI:** 10.1002/jgh3.70341

**Published:** 2026-02-05

**Authors:** Shogo Kitahata, Mai Saito, Yuka Kimura, Ayaka Nakamura, Toru Usui, Kanako Kato, Kei Onishi, Kozue Kanemitsu‐Okada, Tomoe Kawamura, Hideko Ohama, Taira Kuroda, Junko Matsuoka, Fujimasa Tada, Hideki Miyata, Atsushi Hiraoka, Eiji Tsubouchi, Tomoyuki Ninomiya, Yoichi Hiasa

**Affiliations:** ^1^ Gastroenterology Center, Ehime Prefectural Central Hospital Matsuyama Ehime Japan; ^2^ Department of Gastroenterology and Metabology Ehime University Graduate School of Medicine Matsuyama Ehime Japan

**Keywords:** endoscopic activity, fecal biomarkers, leucine‐rich α2‐glycoprotein, ulcerative colitis

## Abstract

**Aims:**

To determine whether the performance of leucine‐rich α2‐glycoprotein (LRG) as a biomarker for disease activity in patients with ulcerative colitis (UC) may depend on the extent of the disease.

**Methods and Results:**

We evaluated the correlation between various biomarkers, including the serum biomarker LRG, and endoscopic activity in 171 patients with UC. Patients were categorized into two groups based on disease extent: patients with pancolitis and those with left‐sided colitis and proctitis. LRG demonstrated similar diagnostic accuracy compared to fecal markers in patients with UC exhibiting pancolitis (area under the curve [AUC] for predicting endoscopic mucosal healing: LRG, 0.92; fecal immunochemical testing [FIT], 0.95; and fecal calprotectin [Fcal], 0.96) (FIT vs. LRG, *p* = 0.886; Fcal vs. LRG, *p* = 0.412). Conversely, for patients with left‐sided colitis and proctitis, fecal markers outperformed LRG in predicting endoscopic mucosal healing (AUC: LRG, 0.66; FIT, 0.94; and Fcal, 0.92) (FIT vs. LRG, *p* < 0.001; Fcal vs. LRG, *p* = 0.004).

**Conclusion:**

Our results support selecting biomarkers according to disease extent for the management of inflammatory bowel disease. In patients with UC and pancolitis, LRG is a viable alternative when fecal samples are not feasible. Conversely, for patients with left‐sided colitis and proctitis, fecal markers with high diagnostic accuracy are recommended.

## 1 Introduction

The incidence of ulcerative colitis (UC), an inflammatory bowel disease, is increasing worldwide [1]. Recent advances in medicine have led to the development of new biomarkers for assessing disease activity. Fecal biomarkers, including fecal calprotectin (Fcal), and screening techniques, including fecal immunochemical testing (FIT), are valuable tools for assessing disease activity in patients with UC [2, 3, 4, 5]. However, obtaining fecal specimens on demand can be difficult, making their use in routine clinical care cumbersome. The performance of fecal markers in predicting disease activity varies depending on disease extent, particularly in left‐sided colitis and proctitis compared to pancolitis [6]. C‐reactive protein (CRP), one of the most commonly used blood markers [7, 8], is relatively simple to obtain but may not be elevated in all patients with active disease [9]. A blood biomarker, leucine‐rich α2‐glycoprotein (LRG) [10, 11, 12, 13, 14, 15, 16], reportedly reflects inflammation more sensitively than CRP [17, 18, 19]. Moreover, LRG may have greater utility than other biomarkers, as it can be detected and quantified from a blood sample, enabling simple, rapid, and reproducible screening. Herein, we hypothesized that the performance of LRG as a marker for disease activity differs between patients with left‐sided colitis and proctitis versus those with pancolitis. Few studies have compared the performance of LRG and other biomarkers according to disease extent. Particularly, the impact of disease extent on the association between endoscopic mucosal healing and LRG in patients with UC remains unknown. In this study, we aimed to investigate the effect of disease extent on LRG as a marker for endoscopic mucosal healing in patients with UC.

## 2 Methods

### 2.1 Participants

Biomarkers were quantified simultaneously with colonoscopy in 180 patients with UC who attended Ehime Prefectural Central Hospital (Ehime, Japan) between July 2021 and March 2023. The diagnosis of UC was based on established clinical, radiographic, endoscopic, and histopathologic criteria [20]. Mayo endoscopic subscores (MES) were obtained from all patients, and serum and fecal samples were collected. Additionally, LRG, FIT, and Fcal levels were measured within 2 months of colonoscopy. Some patients with longer intervals between colonoscopy and biomarker measurement were included if they remained clinically stable during this period. Exclusion criteria included previous colostomy or ileostomy and the presence of conditions that could affect LRG and CRP levels such as infections, malignancies, or extraintestinal complications. Ultimately, 171 patients were enrolled in this cross‐sectional study. These patients with UC were categorized into two groups: those with pancolitis and those with left‐sided colitis and proctitis (Figure S1).

### 2.2 Evaluation of UC Disease Activity

Endoscopic disease activity was assessed by a gastroenterologist using the MES, with a score of ≤ 1 defined as endoscopic mucosal healing [21]. The MES was scored by two physicians with at least 5 years of clinical experience in endoscopy. Based on medical history and endoscopic findings at inclusion, patients were classified according to the Montreal Classification as E1 (proctitis), E2 (left‐sided colitis), or E3 (pancolitis), reflecting the extent of disease [22].

### 2.3 Biomarker Measurements

LRG levels were measured using a commercially available kit, Nanopia LRG (Sekisui Medical Company Ltd., Tokyo, Japan). CRP levels were determined through routine laboratory analysis. Fecal samples were collected within 2 days of biomarker measurement and stored at −20°C until analysis. Fecal calprotectin was quantified using a fluorescent enzyme immunoassay with the Phadia EliATM Calprotectin 2 kit (Thermo Fisher Scientific, Phadia AB, Uppsala, Sweden). Sample preparation utilized a hemodia sampling probe (Eiken Chemical, Tokyo, Japan). Fecal hemoglobin levels were quantified using the OC‐Sensor DIANA system (Eiken Chemical), which detects concentrations between 20 and 1000 ng/mL. Samples exceeding 1000 ng/mL were appropriately diluted prior to remeasurement.

### 2.4 Statistical Analysis

All analyses were conducted using R software (version 3.5.0; R Software for Statistical Computing, Vienna, Austria). Fisher's exact test was used for nominal variables, and the Wilcoxon rank‐sum test for continuous variables. Correlations were assessed via Spearman's rank correlation coefficient. The diagnostic performance of biomarkers for detecting UC disease activity was evaluated using receiver operating characteristic (ROC) curves, and the area under the curve (AUC) was calculated. Delong's test was used to compare AUC values. Logistic regression analyses estimated crude odds ratios (ORs) with 95% confidence intervals (CIs) for endoscopic mucosal healing in relation to biomarker levels. Multiple logistic regression models adjusted for potential confounders selected a priori: age, sex, use of 5‐aminosalicylic acid, and use of prednisolone. Statistical significance was set at *p* < 0.05.

## 3 Results

### 3.1 Clinical Characteristics of the Patients

Of the 171 patients with UC, 83 (48.5%) had pancolitis and 88 (51.5%) had left‐sided colitis and proctitis (Figure S1). The clinical characteristics of patients with UC and pancolitis or with left‐sided colitis and proctitis are summarized in Table 1. The rates of endoscopic mucosal healing were 54.2% in the pancolitis group and 59.0% in the left‐sided colitis and proctitis group. Patients with left‐sided colitis and proctitis were older than those with pancolitis, but no significant differences were observed between the two groups in terms of sex, frequency of endoscopic mucosal healing, or treatment (Table 1).

**TABLE 1:** Characteristics of patients with UC with either pancolitis or left‐sided colitis and proctitis.

Demographic and treatment characteristics	UC (*n* = 83)	UC (*n* = 88)	*p*
	Pancolitis	Left‐sided colitis and proctitis	
Age, years^a^	50.0 (35–57)	55.5 (41–62)	0.038
Sex, male/female	47/36	43/45	0.359
Treatment			
5‐Aminosalicylic acid, *n* (%)	67 (80.7%)	78 (88.6%)	0.201
Corticosteroids, *n* (%)	7 (8.4%)	5 (5.6%)	0.558
Immunomodulators, *n* (%)	18 (21.7%)	17 (19.3%)	0.707
Biologic agents, *n* (%)	18 (21.7%)	14 (15.9%)	0.433
Calcineurin inhibitors, *n* (%)	1 (1.2%)	0	0.485
Probiotics, *n* (%)	22 (26.5%)	26 (29.5%)	0.734
Antibiotics, *n* (%)	0	0	
Endoscopic mucosal healing (MES ≤ 1), *n* (%)	45 (54.2%)	52 (59.0%)	0.540

*Note:* A Wilcoxon rank‐sum test was performed to compare the ages of patients with UC and pancolitis with those with left‐sided colitis and proctitis. A Fisher's exact test was performed to compare the sex distribution, treatment, and endoscopic mucosal healing frequency between patients with UC and pancolitis with those with left‐sided colitis and proctitis.

Abbreviations: MES, Mayo endoscopic subscore; UC, ulcerative colitis.

^a^ Median (interquartile range).

### 3.2 Correlations Between Endoscopic Mucosal Healing and Biomarkers of UC in Patients With Pancolitis and Those With Left‐Sided Colitis and Proctitis

**FIGURE 1:**
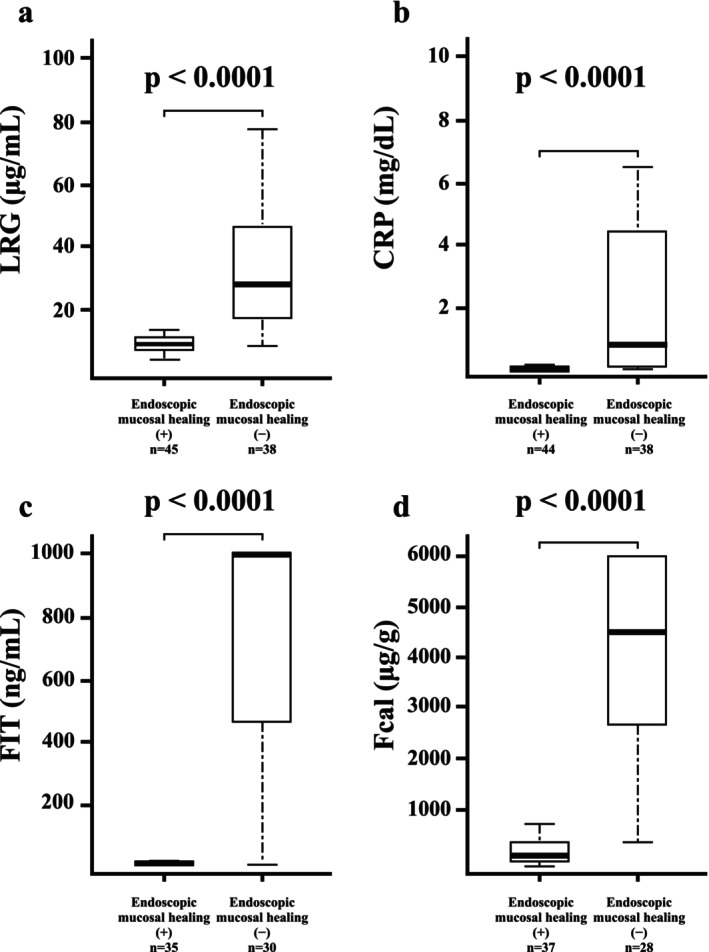
Correlation between endoscopic mucosal healing and biomarkers in patients with ulcerative colitis with pancolitis. (a) Leucine‐rich α2‐glycoprotein (LRG), (b) C‐reactive protein (CRP), (c) fecal immunochemical testing (FIT), and (d) fecal calprotectin (Fcal).

**FIGURE 2:**
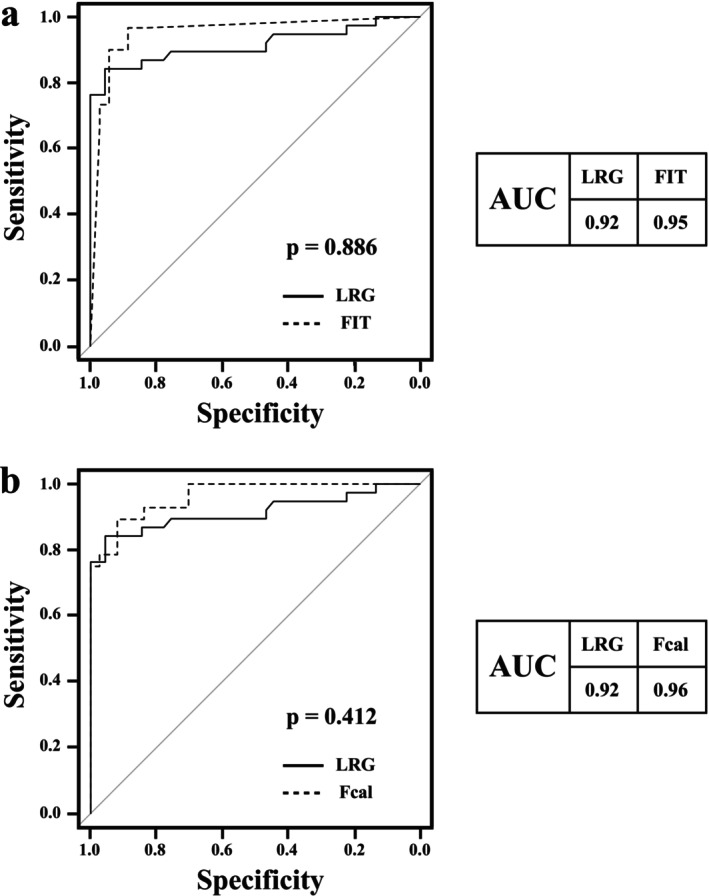
Receiver operating characteristic (ROC) curves for leucine‐rich α2‐glycoprotein (LRG), fecal immunochemical testing (FIT), and fecal calprotectin (Fcal) in evaluating endoscopic mucosal healing in patients with ulcerative colitis with pancolitis. Area under the ROC curve (AUC) values are indicated. The diagonal line represents random classification (AUC = 0.5).

All biomarkers were significantly elevated in patients with UC and pancolitis and active disease (LRG, *p* < 0.0001; CRP, *p* < 0.0001; FIT, *p* < 0.0001; and Fcal, *p* < 0.0001; Figure 1). In patients with UC and pancolitis, the AUCs for predicting endoscopic mucosal healing were 0.92 for LRG, 0.89 for CRP, 0.95 for FIT, and 0.96 for Fcal. The sensitivity and specificity of LRG, CRP, FIT, and Fcal were 0.84 and 0.96; 0.87 and 0.79; 0.97 and 0.89; and 0.89 and 0.92, respectively. The optimal cutoff values were < 16.1 μg/mL for LRG, < 0.1 mg/dL for CRP, < 73 ng/mL for FIT, and < 868 μg/g for Fcal (Table 2). The AUC for LRG was comparable to those of FIT and Fcal (FIT vs. LRG, *p* = 0.886; Fcal vs. LRG, *p* = 0.412; Figure 2).

**TABLE 2:** Predictive values, sensitivity, and specificity of biomarkers for endoscopic mucosal healing in patients with UC and pancolitis.

Biomarkers	AUC	Sensitivity	Specificity	PPV	NPV	Cutoff
LRG	0.92	0.84	0.96	0.94	0.88	< 16.1 μg/mL
CRP	0.89	0.87	0.79	0.79	0.87	< 0.1 mg/dL
FIT	0.95	0.97	0.89	0.88	0.97	< 73 ng/mL
Fcal	0.96	0.89	0.92	0.89	0.92	< 868 μg/g

Abbreviations: AUC, area under curve; CRP, C‐reactive protein; Fcal, fecal calprotectin; FIT, fecal immunochemical testing; LRG, leucine‐rich α2‐glycoprotein; NPV, negative predictive value; PPV, positive predictive value; UC, ulcerative colitis.

**FIGURE 3:**
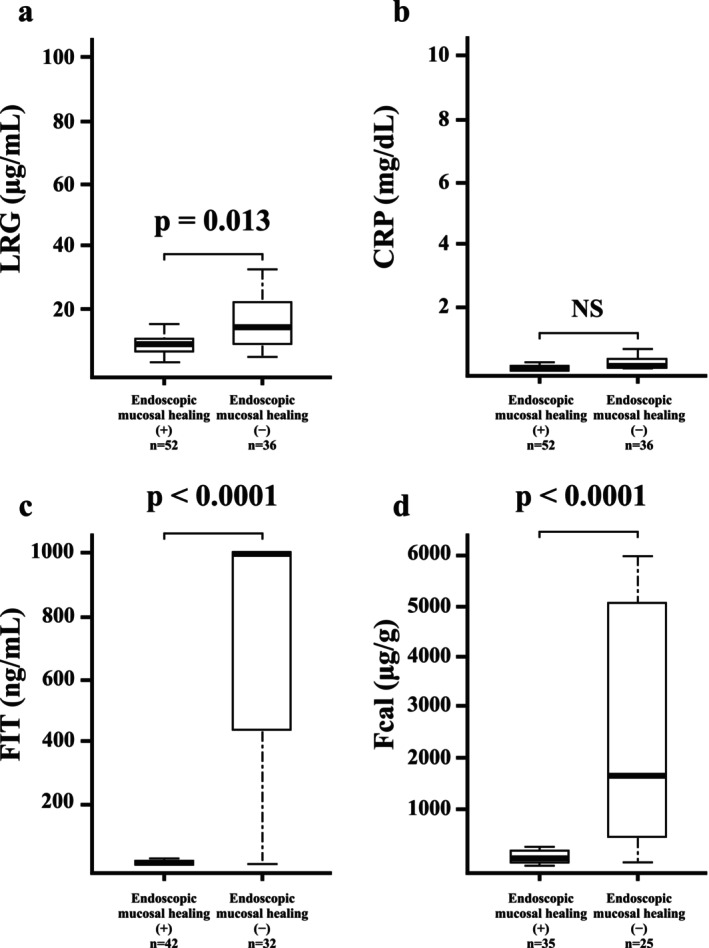
Correlation between endoscopic mucosal healing and biomarkers in patients with ulcerative colitis with left‐sided colitis and proctitis. (a) Leucine‐rich α2‐glycoprotein (LRG), (b) C‐reactive protein (CRP), (c) fecal immunochemical testing (FIT), and (d) fecal calprotectin (Fcal).

**FIGURE 4:**
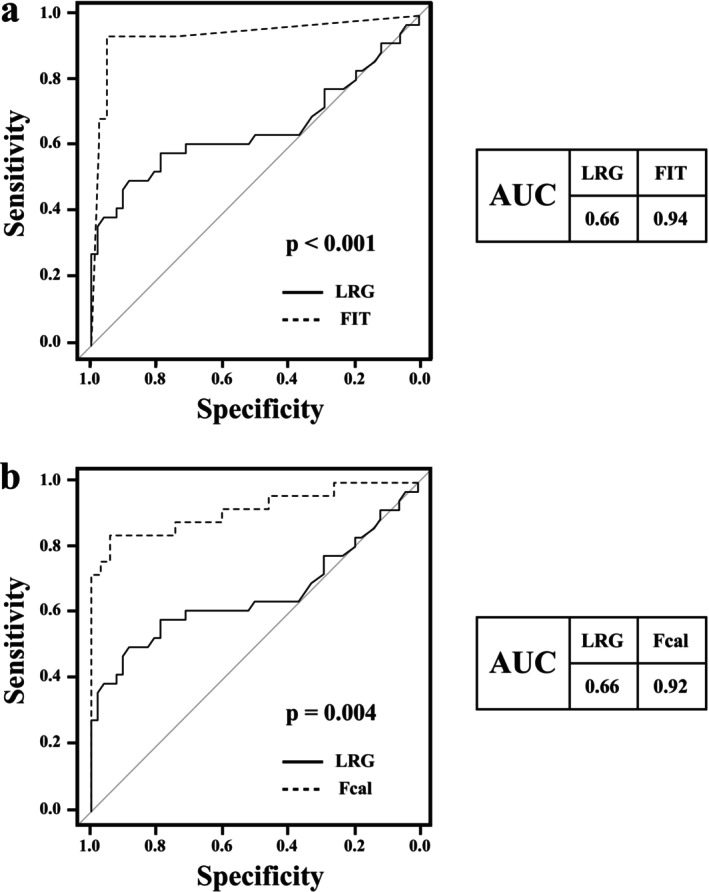
Receiver operating characteristic (ROC) curves for leucine‐rich α2‐glycoprotein (LRG), fecal immunochemical testing (FIT), and fecal calprotectin (Fcal) in evaluating endoscopic mucosal healing in patients with ulcerative colitis with left‐sided colitis and proctitis. Area under the ROC curve (AUC) values are shown. The diagonal line represents random classification (AUC = 0.5).

In patients with left‐sided colitis and proctitis, LRG and fecal markers were significantly elevated in those with active disease, whereas CRP was not significantly elevated (LRG, *p* = 0.013; CRP, *p* = 0.054; FIT, *p* < 0.0001; and Fcal, *p* < 0.0001; Figure 3). The AUCs for predicting endoscopic mucosal healing were 0.66 for LRG, 0.62 for CRP, 0.94 for FIT, and 0.92 for Fcal. The sensitivity and specificity of LRG, CRP, FIT, and Fcal were 0.50 and 0.89; 0.39 and 0.85; 0.94 and 0.95; and 0.84 and 0.94, respectively. The corresponding cutoff values were < 14.9 μg/mL for LRG, < 0.14 mg/dL for CRP, < 231 ng/mL for FIT, and < 329 μg/g for Fcal (Table 3). The AUCs of FIT and Fcal were significantly higher than those of LRG (FIT vs. LRG, *p* < 0.001; Fcal vs. LRG, *p* = 0.004; Figure 4).

**TABLE 3:** Endoscopic mucosal healing biomarker predictive values, sensitivities, and specificities for patients with UC with left‐sided colitis and proctitis.

Biomarkers	AUC	Sensitivity	Specificity	PPV	NPV	Cutoff
LRG	0.66	0.50	0.89	0.75	0.72	< 14.9 μg/mL
CRP	0.62	0.39	0.85	0.64	0.67	< 0.14 mg/dL
FIT	0.94	0.94	0.95	0.94	0.95	< 231 ng/mL
Fcal	0.92	0.84	0.94	0.91	0.89	< 329 μg/g

Abbreviations: AUC, area under curve; CRP, C‐reactive protein; Fcal, fecal calprotectin; FIT, fecal immunochemical testing; LRG, leucine‐rich α2‐glycoprotein; NPV, negative predictive value; PPV, positive predictive value; UC, ulcerative colitis.

### 3.3 Subanalysis of Patients With Proctitis

To further investigate whether the reduced diagnostic performance of LRG according to disease extent was driven specifically by proctitis, we performed a subanalysis restricted to patients with proctitis (*n* = 44). In this subgroup, fecal marker levels were significantly elevated in those with active disease, whereas LRG and CRP levels were not significantly elevated (LRG, *p* = 0.585; CRP, *p* = 0.167; FIT, *p* = 0.001; Fcal, *p* = 0.003; Figure S2). The AUC of LRG for predicting endoscopic mucosal healing was 0.44, with a sensitivity of 0.30 and specificity of 0.94, using a cutoff of < 15.8 μg/mL (Table S1). In contrast, fecal biomarkers retained high diagnostic performance (FIT: AUC = 0.83; Fcal: AUC = 0.85). Although these AUCs differed, the pairwise comparisons did not reach statistical significance (*p* > 0.05), likely reflecting the limited sample size (Figure S3).

### 3.4 Complete Endoscopic Mucosal Healing (MES 0) Subanalysis

We also examined biomarker performance using the stricter definition of complete endoscopic mucosal healing (MES 0) to enhance the clinical interpretability. The rates of complete endoscopic mucosal healing were 40.9% in the pancolitis group and 36.4% in the left‐sided colitis and proctitis group. In patients with pancolitis, LRG showed good diagnostic accuracy (AUC = 0.81), while FIT and Fcal demonstrated high accuracy (AUC = 0.82 and 0.93, respectively). In patients with left‐sided colitis and proctitis, LRG showed limited discriminative performance (AUC = 0.58), whereas fecal biomarkers maintained high accuracy (FIT: 0.86; Fcal: 0.85). The ROC curves and diagnostic characteristics for MES 0 are presented in Figures S4–S7 and Tables S2 and S3.

## 4 Discussion

This study revealed that the discriminative performance of various biomarkers for detecting disease activity in UC varied according to disease extent. In patients with UC and pancolitis, LRG was comparable to FIT and Fcal in predicting endoscopic mucosal healing. By contrast, FIT and Fcal showed superior performance to LRG in predicting endoscopic mucosal healing in patients with UC with left‐sided colitis and proctitis. Moreover, our supplementary analysis using the stricter definition of complete mucosal healing (MES 0) also showed trends consistent with the primary findings. To the best of our knowledge, this is the first study to compare the discriminative performance of LRG and fecal markers based on disease extent.

Reports comparing the discriminative performance of LRG with CRP, FIT, and Fcal for disease activity are limited, and the results remain inconsistent [2, 19, 23, 24, 25, 26, 27]. Serada et al. reported that LRG, compared to CRP, was more elevated in patients with clinical UC activity and significantly higher in active disease than in remission [23]. Moreover, LRG has been shown to correlate more closely with endoscopic disease activity than CRP. Some studies have suggested that LRG levels can differentiate between clinical remission and endoscopic mucosal healing, even in patients with normal CRP levels [2]. However, a recent study found no significant difference in the correlation of LRG and CRP with UC disease activity [25]. Additionally, LRG has been associated with clinical and endoscopic outcomes in patients treated with adalimumab and demonstrated better correlation with endoscopic activity than Fcal [26]. Conversely, a previous work stated that although LRG correlates with endoscopic disease activity, it performs less effectively than fecal markers [2]. These discrepancies may be partly explained by differences in sample size, patient demographics (e.g., age and sex), and definitions of clinical remission and endoscopic mucosal healing.

Our study has several strengths compared with previous reports. First, we assessed endoscopic scores and simultaneously measured LRG, FIT, and Fcal levels. Second, we evaluated how the characteristics of each biomarker may differ by disease extent. Fecal markers directly detect neutrophil and blood components as stool passes through the affected area; therefore, they are sensitive to inflammation in the sigmoid colon and rectum. Previous reports have suggested that Fcal levels in left‐sided colitis and proctitis may be higher than those in pancolitis in histologically active disease [28]. LRG is induced by interleukin (IL)‐6 and other pro‐inflammatory cytokines, such as IL‐22 and tumor necrosis factor [2]. Although the serum marker LRG is also produced locally by intestinal epithelial cells and infiltrating neutrophils, it primarily reflects the systemic inflammatory response and may be affected by the disease extent, which is the source of inflammatory cytokine production. Our study suggests that in UC and pancolitis, the total amount of inflammatory cytokines produced is high, making LRG a good indicator of inflammation, whereas in UC with left‐sided colitis and proctitis, the extent of inflammation is small, making it difficult for LRG blood levels to reflect inflammation. These findings support the use of appropriate biomarkers based on disease extent for the treatment of inflammatory bowel disease. In patients with UC and pancolitis, LRG is a viable alternative when fecal samples are not feasible. Conversely, fecal markers with high diagnostic performance may be recommended for patients with left‐sided colitis and proctitis. Furthermore, the optimal cutoff value for LRG differed between patients with pancolitis and those with left‐sided colitis and proctitis. Our ROC‐derived threshold was slightly higher in pancolitis cases (< 16.1 μg/mL) and lower in left‐sided colitis and proctitis cases (< 14.9 μg/mL). Previous studies have reported ranges for LRG cutoff values of approximately 12–14 μg/mL for predicting endoscopic mucosal healing [2, 15]. The higher cutoff value for LRG in defining endoscopic mucosal healing, in contrast to those in earlier reports, can be attributed to factors such as endoscopic mucosal healing rates and the specific definition used. Importantly, the disease extent may influence the optimal LRG cutoff values, which should be considered in the biomarker interpretation.

Nonetheless, this study has some limitations. First, this was a single‐center study with a relatively small sample size, and the findings may not be generalizable to all Japanese patients with UC. However, our sample size was calculated based on previous reports evaluating biomarker performance [6, 19, 25]. Second, we did not fully assess the potential effects of comorbidities that might elevate LRG levels, such as asymptomatic viral infections or arthritis. Third, in this study, the inclusion of mainly mild‐to‐moderate cases may have underestimated the elevated LRG levels observed in more severe UC. Additionally, since biomarker measurements were taken within 2 months of colonoscopy, the possible influence of disease activity fluctuations should be acknowledged. Therefore, a prospective study including severe cases is warranted to explore the association between the disease extent and the diagnostic performance of LRG in UC.

In conclusion, fecal markers were superior to LRG and CRP in predicting endoscopic mucosal healing in patients with left‐sided colitis and proctitis; however, LRG was comparable to fecal markers in patients with pancolitis. The use of biomarkers selected according to disease extent may improve the accuracy of endoscopic activity assessment and offer clinical utility in the management of UC. Therefore, UC management strategies should be tailored according to the disease extent using both serum and fecal biomarkers.

## Funding

The authors have nothing to report.

## Ethics Statement

The study protocol was developed in accordance with the 1964 Declaration of Helsinki and subsequent versions of the ethical guidelines. This study protocol was reviewed and approved by the Institutional Ethics Committee of Ehime Prefectural Central Hospital (approval no. 03‐57). All authors reviewed and approved the manuscript.

## Consent

All participants provided written informed consent.

## Conflicts of Interest

The authors declare no conflicts of interest.

## Supporting information


**Data S1:** Supporting Information.

## Data Availability

The data that support the findings of this study are not publicly available due to privacy reasons but are available from the corresponding author upon request.

## References

[1] G. G. Kaplan , “The Global Burden of IBD: From 2015 to 2025,” Nature Reviews. Gastroenterology & Hepatology 12 (2015): 720–727. 26323879 10.1038/nrgastro.2015.150

[2] E. Yasutomi , T. Inokuchi , S. Hiraoka , et al., “Leucine‐Rich Alpha‐2 Glycoprotein as a Marker of Mucosal Healing in Inflammatory Bowel Disease,” Scientific Reports 11 (2021): 11086. 34045529 10.1038/s41598-021-90441-x PMC8160157

[3] S. Takashima , J. Kato , S. Hiraoka , et al., “Evaluation of Mucosal Healing in Ulcerative Colitis by Fecal Calprotectin vs. Fecal Immunochemical Test,” American Journal of Gastroenterology 110 (2015): 873–880. 25823769 10.1038/ajg.2015.66

[4] S. Hiraoka , T. Inokuchi , A. Nakarai , et al., “Fecal Immunochemical Test and Fecal Calprotectin Results Show Different Profiles in Disease Monitoring for Ulcerative Colitis,” Gut Liver 12 (2018): 142–148. 28873508 10.5009/gnl17013 PMC5832338

[5] S. Hiraoka , S. Takashima , T. Inokuchi , et al., “The Novel Latex Agglutination Turbidimetric Immunoassay System for Simultaneous Measurements of Calprotectin and Hemoglobin in Feces,” Intestinal Research 17 (2019): 202–209. 30541228 10.5217/ir.2018.00086 PMC6505093

[6] A. Sakuraba , N. Nemoto , N. Hibi , et al., “Extent of Disease Affects the Usefulness of Fecal Biomarkers in Ulcerative Colitis,” BMC Gastroenterology 21 (2021): 197. 33933033 10.1186/s12876-021-01788-4 PMC8088576

[7] C. Toniatti , R. Arcone , B. Majello , U. Ganter , G. Arpaia , and G. Ciliberto , “Regulation of the Human C‐Reactive Protein Gene, a Major Marker of Inflammation and Cancer,” Molecular Biology & Medicine 7 (1990): 199–212. 2170808

[8] S. Vermeire , G. Van Assche , and P. Rutgeerts , “C‐Reactive Protein as a Marker for Inflammatory Bowel Disease,” Inflammatory Bowel Diseases 10 (2004): 661–665. 15472532 10.1097/00054725-200409000-00026

[9] B. E. Sands , “Biomarkers of Inflammation in Inflammatory Bowel Disease,” Gastroenterology 149 (2015): 1275–1285.e2. 26166315 10.1053/j.gastro.2015.07.003

[10] H. Haupt and S. Baudner , “Isolation and Characterization of an Unknown, Leucine‐Rich 3.1‐S‐alpha2‐Glycoprotein From Human Serum (Author's Transl),” Hoppe‐Seyler's Zeitschrift für Physiologische Chemie 358 (1977): 639–646. 69600

[11] N. Takahashi , Y. Takahashi , and F. W. Putnam , “Periodicity of Leucine and Tandem Repetition of a 24‐Amino Acid Segment in the Primary Structure of Leucine‐Rich Alpha 2‐Glycoprotein of Human Serum,” Proceedings of the National Academy of Sciences of the United States of America 82 (1985): 1906–1910. 3856868 10.1073/pnas.82.7.1906 PMC397442

[12] R. Shirai , F. Hirano , N. Ohkura , K. Ikeda , and S. Inoue , “Up‐Regulation of the Expression of Leucine‐Rich Alpha(2)‐Glycoprotein in Hepatocytes by the Mediators of Acute‐Phase Response,” Biochemical and Biophysical Research Communications 382 (2009): 776–779. 19324010 10.1016/j.bbrc.2009.03.104 PMC7092932

[13] M. Fujimoto , S. Serada , K. Suzuki , et al., “Brief Report: Leucine‐Rich α2‐Glycoprotein as a Potential Biomarker for Joint Inflammation During Anti‐Interleukin‐6 Biologic Therapy in Rheumatoid Arthritis,” Arthritis & Rhematology 67 (2015): 2056–2060. 10.1002/art.39164 25917892

[14] S. Matsumoto and H. Mashima , “Clinical Profiles of Leucine‐Rich Alpha‐2 Glycoprotein for Indicating Mucosal Healing in Ulcerative Colitis Patients Under Administration of Molecular‐Targeted Drug,” Digestive Diseases 43 (2025): 11–18. 39462503 10.1159/000542062

[15] Y. Aoyama , S. Hiraoka , E. Yasutomi , et al., “Changes of Leucine‐Rich Alpha 2 Glycoprotein Could Be a Marker of Changes of Endoscopic and Histologic Activity of Ulcerative Colitis,” Scientific Reports 15 (2025): 5248. 39939376 10.1038/s41598-025-89615-8 PMC11822068

[16] T. Amano , T. Yoshihara , S. Shinzaki , et al., “Selection of Anti‐Cytokine Biologics by Pretreatment Levels of Serum Leucine‐Rich Alpha‐2 Glycoprotein in Patients With Inflammatory Bowel Disease,” Scientific Reports 14 (2024): 29755. 39613813 10.1038/s41598-024-80285-6 PMC11607305

[17] S. Vermeire , G. Van Assche , and P. Rutgeerts , “Laboratory Markers in IBD: Useful, Magic, or Unnecessary Toys?,” Gut 55 (2006): 426–431. 16474109 10.1136/gut.2005.069476 PMC1856093

[18] S. Serada , M. Fujimoto , A. Ogata , et al., “iTRAQ‐Based Proteomic Identification of Leucine‐Rich Alpha‐2 Glycoprotein as a Novel Inflammatory Biomarker in Autoimmune Diseases,” Annals of the Rheumatic Diseases 69 (2010): 770–774. 19854709 10.1136/ard.2009.118919

[19] S. Shinzaki , K. Matsuoka , H. Iijima , et al., “Leucine‐Rich Alpha‐2 Glycoprotein Is a Serum Biomarker of Mucosal Healing in Ulcerative Colitis,” Journal of Crohn's & Colitis 11 (2017): 84–91. 10.1093/ecco-jcc/jjw132 PMC5175492 27466171

[20] K. Matsuoka , T. Kobayashi , F. Ueno , et al., “Evidence‐Based Clinical Practice Guidelines for Inflammatory Bowel Disease,” Journal of Gastroenterology 53 (2018): 305–353. 29429045 10.1007/s00535-018-1439-1 PMC5847182

[21] K. W. Schroeder , W. J. Tremaine , and D. M. Ilstrup , “Coated Oral 5‐Aminosalicylic Acid Therapy for Mildly to Moderately Active Ulcerative Colitis. A Randomized Study,” New England Journal of Medicine 317 (1987): 1625–1629. 3317057 10.1056/NEJM198712243172603

[22] J. Satsangi , M. S. Silverberg , S. Vermeire , and J. Colombel , “The Montreal Classification of Inflammatory Bowel Disease: Controversies, Consensus, and Implications,” Gut 55 (2006): 749–753. 16698746 10.1136/gut.2005.082909 PMC1856208

[23] S. Serada , M. Fujimoto , F. Terabe , et al., “Serum Leucine‐Rich Alpha‐2 Glycoprotein Is a Disease Activity Biomarker in Ulcerative Colitis,” Inflammatory Bowel Diseases 18 (2012): 2169–2179. 22374925 10.1002/ibd.22936

[24] N. Nakamura , Y. Honzawa , S. Nishimon , et al., “Combined Serum Albumin, Fecal Immunochemical Test, and Leucine‐Rich Alpha‐2 Glycoprotein Levels for Predicting Prognosis in Remitting Patients With Ulcerative Colitis,” Scientific Reports 13 (2023): 13863. 37620642 10.1038/s41598-023-41137-x PMC10449766

[25] T. Yoshimura , K. Mitsuyama , R. Sakemi , et al., “Evaluation of Serum Leucine‐Rich Alpha‐2 Glycoprotein as a New Inflammatory Biomarker of Inflammatory Bowel Disease,” Mediators of Inflammation 2021 (2021): 8825374. 33623482 10.1155/2021/8825374 PMC7874844

[26] S. Shinzaki , K. Matsuoka , H. Tanaka , et al., “Leucine‐Rich Alpha‐2 Glycoprotein Is a Potential Biomarker to Monitor Disease Activity in Inflammatory Bowel Disease Receiving Adalimumab: PLANET Study,” Journal of Gastroenterology 56 (2021): 560–569. 33942166 10.1007/s00535-021-01793-0 PMC8137624

[27] A. G. Røseth , E. Aadland , and J. Jahnsen , “Assessment of Disease Activity in Ulcerative Colitis by Faecal Calprotectin, a Novel Granulocyte Marker Protein,” Digestion 58 (1997): 176–180. 9144308 10.1159/000201441

[28] N. Ishida , T. Takebe , K. Takahashi , et al., “Optimal Positioning of Biomarkers According to Ulcerative Colitis Activity,” Scientific Reports 15 (2025): 19916. 40481096 10.1038/s41598-025-04908-2 PMC12144207

