# Supplementary material for: Effect of Disease Extent on Leucine‐Rich α2‐Glycoprotein as a Marker for Endoscopic Mucosal Healing in Patients With Ulcerative Colitis
Source: JGH Open. 2026 Feb 5;10(2):e70341. doi: 10.1002/jgh3.70341 (PMC12874191; doi:10.1002/jgh3.70341)
Supplement: Supplementary file 1 — Data S1: Supporting Information. [file JGH3-10-e70341-s001.docx]

***Supporting Information***

***Effect of Disease Extent on Leucine-Rich α2-Glycoprotein as a Marker for Endoscopic Mucosal Healing in Patients with Ulcerative Colitis***

Shogo Kitahata^a^, Mai Saito^a^, Yuka Kimura^a^, Ayaka Nakamura^a^, Toru Usui^a^, Kanako Kato^a^, Kei Onishi^a^, Kozue Kanemitsu-Okada^a^, Tomoe Kawamura^a^, Hideko Ohama^a^, Taira Kuroda^a^, Junko Matsuoka^a^, Fujimasa Tada^a^, Hideki Miyata^a^, Atsushi Hiraoka^a^, Eiji Tsubouchi^a^,Tomoyuki Ninomiya^a^, Yoichi Hiasa^b^

^a^ Gastroenterology Center, Ehime Prefectural Central Hospital, Ehime, Japan

^b^ Department of Gastroenterology and Metabology, Ehime University Graduate School of Medicine, Ehime, Japan

Supplementary Fig. 1. Patient flowchart of the study.

Supplementary Fig. 2. Correlation between endoscopic mucosal healing and biomarkers in patients with ulcerative colitis with proctitis. (a) Leucine-rich α2-glycoprotein (LRG), (b) C-reactive protein (CRP), (c) fecal immunochemical testing (FIT), and (d) fecal calprotectin (Fcal).

Supplementary Fig. 3. Receiver operating characteristic (ROC) curves for leucine-rich α2-glycoprotein (LRG), fecal immunochemical testing (FIT), and fecal calprotectin (Fcal) in evaluating endoscopic mucosal healing in patients with ulcerative colitis with proctitis. Area under the ROC curve (AUC) values are shown. The diagonal line represents random classification (AUC = 0.5).

Supplementary Fig. 4. Correlation between complete endoscopic mucosal healing (Mayo endoscopic subscore 0) and biomarkers in patients with ulcerative colitis with pancolitis. (a) Leucine-rich α2-glycoprotein (LRG), (b) C-reactive protein (CRP), (c) fecal immunochemical testing (FIT), and (d) fecal calprotectin (Fcal).

Supplementary Fig. 5. Receiver operating characteristic (ROC) curves for leucine-rich α2-glycoprotein (LRG), fecal immunochemical testing (FIT), and fecal calprotectin (Fcal) in evaluating complete endoscopic mucosal healing (Mayo endoscopic subscore 0) in patients with ulcerative colitis with pancolitis. Area under the ROC curve (AUC) values are shown. The diagonal line represents random classification (AUC = 0.5).

Supplementary Fig. 6. Correlation between complete endoscopic mucosal healing (Mayo endoscopic subscore 0) and biomarkers in patients with ulcerative colitis with left-sided colitis and proctitis. (a) Leucine-rich α2-glycoprotein (LRG), (b) C-reactive protein (CRP), (c) fecal immunochemical testing (FIT), and (d) fecal calprotectin (Fcal).

Supplementary Fig. 7. Receiver operating characteristic (ROC) curves for leucine-rich α2-glycoprotein (LRG), fecal immunochemical testing (FIT), and fecal calprotectin (Fcal) in evaluating complete endoscopic mucosal healing (Mayo endoscopic subscore 0) in patients with ulcerative colitis with left-sided colitis and proctitis. Area under the ROC curve (AUC) values are shown. The diagonal line represents random classification (AUC = 0.5).

Supplementary Table 1. Endoscopic mucosal healing biomarker predictive values, sensitivities, and specificities for patients with UC with proctitis.

| **Biomarkers** | **AUC** | **Sensitivity** | **Specificity** | **PPV** | **NPV** | **Cutoff** |
| --- | --- | --- | --- | --- | --- | --- |
| LRG | 0.44 | 0.30 | 0.94 | 0.60 | 0.82 | <15.8 μg/mL |
| CRP | 0.65 | 0.50 | 0.77 | 0.39 | 0.84 | <0.09 mg/dL |
| FIT | 0.83 | 0.78 | 0.96 | 0.88 | 0.93 | <236 ng/mL |
| Fcal | 0.85 | 0.75 | 0.96 | 0.86 | 0.92 | <457 μg/g |

AUC, area under curve; CRP, C-reactive protein; Fcal, fecal calprotectin; FIT, fecal immunochemical testing; LRG, leucine-rich α2-glycoprotein; PPV, positive predictive value; NPV, negative predictive value; UC, ulcerative colitis.

Supplementary Table 2. Predictive values, sensitivity, and specificity of biomarkers for complete endoscopic mucosal healing (MES 0) in patients with UC with pancolitis.

| **Biomarkers** | **AUC** | **Sensitivity** | **Specificity** | **PPV** | **NPV** | **Cutoff** |
| --- | --- | --- | --- | --- | --- | --- |
| LRG | 0.81 | 0.67 | 0.97 | 0.97 | 0.67 | <16.1 μg/mL |
| CRP | 0.77 | 0.71 | 0.79 | 0.83 | 0.65 | <0.1 mg/dL |
| FIT | 0.82 | 0.76 | 0.83 | 0.89 | 0.67 | <24 ng/mL |
| Fcal | 0.93 | 0.92 | 0.77 | 0.86 | 0.87 | <125 μg/g |

AUC, area under curve; CRP, C-reactive protein; Fcal, fecal calprotectin; FIT, fecal immunochemical testing; LRG, leucine-rich α2-glycoprotein; PPV, positive predictive value; NPV, negative predictive value; UC, ulcerative colitis.

Supplementary Table 3. Complete endoscopic mucosal healing (MES 0) biomarker predictive values, sensitivities, and specificities for patients with UC with left-sided colitis and proctitis.

| **Biomarkers** | **AUC** | **Sensitivity** | **Specificity** | **PPV** | **NPV** | **Cutoff** |
| --- | --- | --- | --- | --- | --- | --- |
| LRG | 0.58 | 0.34 | 0.84 | 0.79 | 0.42 | <14.9 μg/mL |
| CRP | 0.52 | 0.30 | 0.84 | 0.77 | 0.41 | <0.14 mg/dL |
| FIT | 0.86 | 0.67 | 1.00 | 1.00 | 0.61 | <199 ng/mL |
| Fcal | 0.85 | 0.72 | 0.86 | 0.90 | 0.62 | <163 μg/g |

AUC, area under curve; CRP, C-reactive protein; Fcal, fecal calprotectin; FIT, fecal immunochemical testing; LRG, leucine-rich α2-glycoprotein; PPV, positive predictive value; NPV, negative predictive value; UC, ulcerative colitis.
